# Lactate Contribution to the Tumor Microenvironment: Mechanisms, Effects on Immune Cells and Therapeutic Relevance

**DOI:** 10.3389/fimmu.2016.00052

**Published:** 2016-02-16

**Authors:** Susana Romero-Garcia, María Maximina B. Moreno-Altamirano, Heriberto Prado-Garcia, Francisco Javier Sánchez-García

**Affiliations:** ^1^Chronic-Degenerative Department, National Institute of Respiratory Diseases “Ismael Cosio Villegas”, Mexico City, Mexico; ^2^Laboratorio de Inmunorregulación, Departamento de Inmunología, Escuela Nacional de Ciencias Biológicas, Instituto Politécnico Nacional, Mexico City, Mexico

**Keywords:** l-lactate metabolism, tumor microenvironment, Warburg effect, monocarboxylate transporter, immune escape

## Abstract

Malignant transformation of cells leads to enhanced glucose uptake and the conversion of a larger fraction of pyruvate into lactate, even under normoxic conditions; this phenomenon of aerobic glycolysis is largely known as the Warburg effect. This metabolic reprograming serves to generate biosynthetic precursors, thus facilitating the survival of rapidly proliferating malignant cells. Extracellular lactate directs the metabolic reprograming of tumor cells, thereby serving as an additional selective pressure. Besides tumor cells, stromal cells are another source of lactate production in the tumor microenvironment, whose role in both tumor growth and the antitumor immune response is the subject of intense research. In this review, we provide an integral perspective of the relationship between lactate and the overall tumor microenvironment, from lactate structure to metabolic pathways for its synthesis, receptors, signaling pathways, lactate-producing cells, lactate-responding cells, and how all contribute to the tumor outcome. We discuss the role of lactate as an immunosuppressor molecule that contributes to tumor evasion and we explore the possibility of targeting lactate metabolism for cancer treatment, as well as of using lactate as a prognostic biomarker.

## Lactate: The Metabolite and Its Synthesis Pathways

The discovery of lactic acid can be traced back to 1789 when, according to Robergs et al. (1), Carl Wilhelm isolated an acid from sour milk samples, opening a whole new research field.

Lactate (2-hydroxypropanoic acid) is a 3-carbon hydroxycarboxylic acid that may exist as two stereoisomers, d-lactate and l-lactate, the latter being the predominant physiological enantiomer. Lactic acid has the formula CH_3_CH(OH)CO_2_H. The anion lactate ${\text{CH}}_{\text{3}}\text{CH}(\text{OH}){\text{CO}}_{2}^{-}$ is the predominant moiety present in the human body, as the p*K*_a_ of the lactate/lactic acid pair is 3.8 (2).

Lactate is produced by most tissues in the human body with the highest levels of production found in muscles. Under healthy conditions, lactate is cleared by the liver and to a lesser extent by the kidneys (3, 4). At a systemic level, lactic acid can serve as a source of energy by being carried to the liver and reconverted into glucose via the Cori cycle (5).

Pyruvate is the final product of glycolysis yielding two moles of ATP for each molecule of glucose in the process. Under normoxic conditions, pyruvate is converted by the enzyme pyruvate dehydrogenase (PDH) into acetyl-CoA, which enters into the tricarboxylic acid (TCA) cycle or Krebs cycle. Under anaerobic conditions, pyruvate is converted to lactic acid by the enzyme lactate dehydrogenase (LDH) (6).

The sequence of the enzymatic reactions that take place during glycolysis was described in 1940 by Embden, Meyerhof, and Parnas, and, since then, “lactate has regularly been vilified as a useless and frequently toxic end product of anaerobic glycolysis,” as stated by Scurr and Gozal (7). It has emerged since long ago that lactate is not a waste metabolic byproduct at all but rather a bioenergetic substrate. More recently, lactate has been regarded as a metabolite with signaling properties and important biological functions, which are out of the scope of the present review.

Tumor cells preferentially convert pyruvate into lactate instead of entering into the TCA cycle, even under normoxic conditions, i.e., by aerobic glycolysis (8). In addition, glutaminolysis can also generate lactate, whereby glutamine is converted to glutamate and then to α-ketoglutarate, followed by the conversion of α-ketoglutarate into malate, which is then oxidized into pyruvate in the cytosol and finally, pyruvate is reduced by LDH-A, producing lactate and NAD^+^ (9, 10). Interestingly, LDH-A is induced by a variety of oncogenes, including *c-myc*, thus linking the malignant transformation at the genetic level with the metabolic pathways leading to lactate production (11).

**Figure 1:**
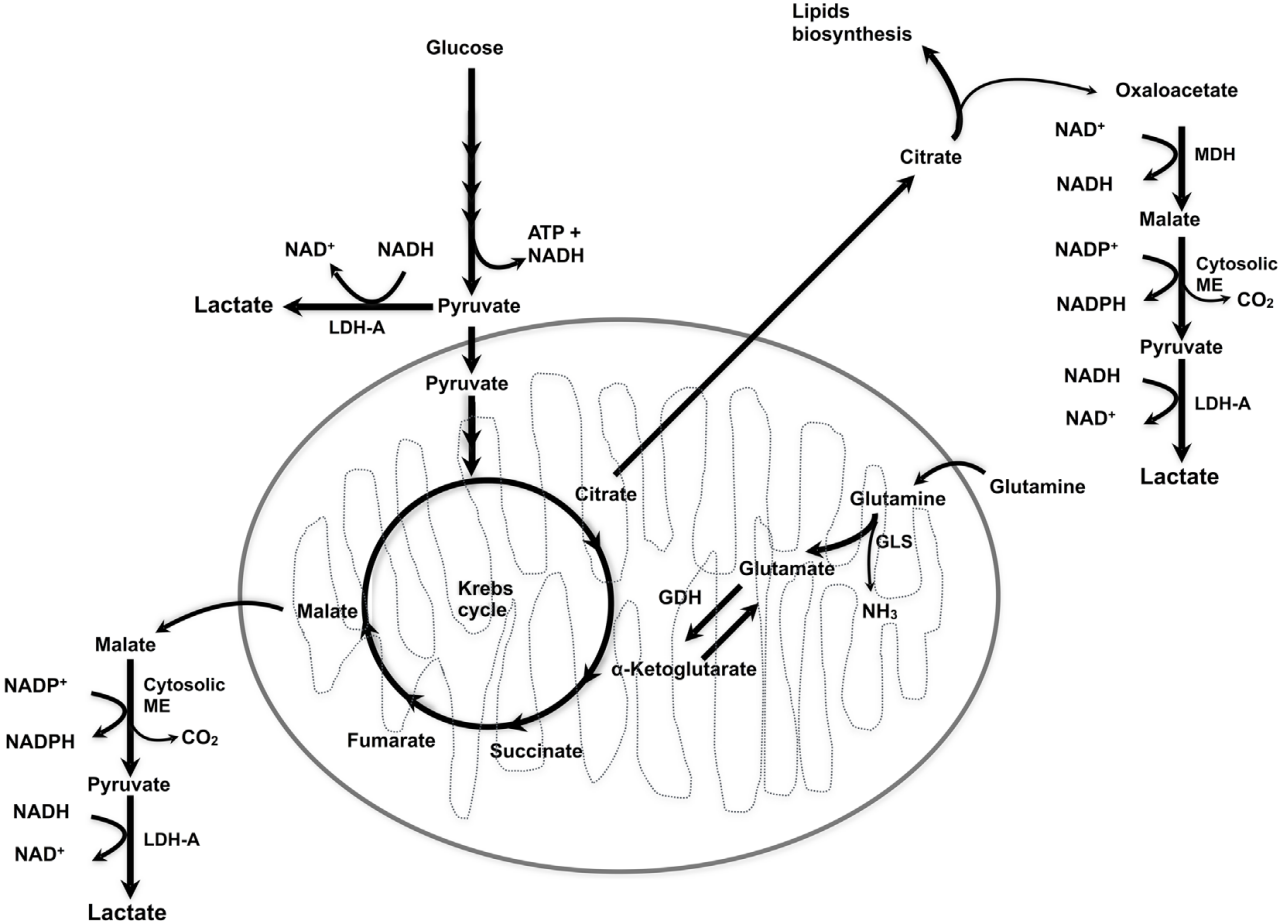
**Lactate synthesis**. ME, malic enzyme; MDH, malate dehydrogenase; LDH-A, lactate dehydrogenase A; GLS, glutaminase; GDH, glutamate dehydrogenase.

The conversion of glutamate to α-ketoglutarate occurs either through oxidative deamination by glutamate dehydrogenase (GDH) in the mitochondrion or by transamination to produce non-essential amino acids in either the cytosol or the mitochondrion. There is some correlation between glucose availability and the use of glutamine as a source of α-ketoglutarate for feeding of the TCA cycle (Figure 1). During intense glucose metabolism, the transamination pathway predominates. On the contrary, when glucose is scarce, GDH becomes the main pathway to supply glutamine carbon to the TCA cycle and is required for cell survival (12–14).

Thus, both glucose and glutamine are metabolic substrates for lactate production, glucose is one of the main metabolic substrates, whereas glutamine accounts for more than 20% of the free amino acid pool in plasma (15, 16), and both have metabolically overlapping functions such as NADPH production and redox homeostasis (17). The individual contribution of glucose and glutamine to the extracellular lactate within the tumor as well as whether glutamine-derived lactate contributes to tumor metabolic symbiosis (discussed below) remains to be analyzed.

## Lactate Production by Tumor Cells: The Warburg Effect

In 1927, Otto Warburg described that, metabolically, tumor cells predominantly rely on increased glycolysis, followed by lactic acid fermentation, even under conditions where oxygen is available. Hence the process was dubbed as “aerobic glycolysis” (8, 18). By contrast, untransformed epithelial cells produce about 20% of their daily energy from glycolysis whereas the rest (about 70%) of that energy comes from the Krebs cycle (19).

Metabolic reprograming of tumor cells modifies the metabolic fluxes, restructuring the Krebs cycle and enhancing glycolysis. The enhanced glycolytic carbon flux, in turn, leads to production of high amounts of lactic acid. It is estimated that tumor cells produce up to 40 times more lactic acid than normal cells (20).

## Lactate Production by Immune System Cells

### Innate Immune Cells

Neutrophils, macrophages, and dendritic cells (DCs) are present in the tumor microenvironment (21, 22), and all these cell populations produce lactate at some point. Neutrophils contain few mitochondria and depend mostly on glycolysis for ATP production, whereas resting macrophages preferentially metabolize the up-taken glucose by glycolysis rather than by oxidative phosphorylation (OXPHOS). In activated macrophages, the expression of hexokinase and glucose-6 phosphate dehydrogenase is up-regulated, indicating that a higher rate of the pentose phosphate pathway is achieved upon stimulation (18, 23). There are two types of macrophages, M1 and M2 macrophages, both differ in their metabolism and in their immune function; while M1 (classically activated) macrophages act as a first line of defense against bacterial infections and obtain energy through glycolysis, M2 (alternatively activated) macrophages are involved in tissue repair and wound healing and use oxidative metabolism to fuel their longer-term functions (24). During tumor progression, the macrophage phenotype changes from M1 to M2 (25). Interestingly, the lactic acid produced within the tumor microenvironment signals macrophages for M2-like polarization and, therefore, it is expected that M2-polarized macrophages cease to produce lactate (26).

The metabolic requirements of DCs depend on their differentiation or activation status, and are different for committed progenitors, quiescent, or activated cells (27). In this regard, the differentiation of human monocytes into DCs is dependent on mitochondrial biogenesis (28). Resting DCs use fatty acid oxidation not only to fuel OXPHOS but also to consume glucose; the metabolic pathway taken by glucose in these cells is still unclear (27, 29). Following activation of DCs with Toll-like receptor (TLR) agonists increases glucose uptake and lactate production (27, 30). Moreover, when glycolysis is pharmacologically inhibited, activation of DCs is also inhibited (31). At later stages of TLR activation, DCs rely mostly on Warburg metabolism for their survival (27, 32). Accordingly, it is likely that only activated DCs can be a source of lactate within a tumor.

### Adaptive Immune Cells

Activation of T lymphocytes through TCR- and CD28-mediated costimulation leads to a rapid increase in the expression of the glucose transporter GLUT-1, glucose uptake, and glycolysis (33–35). An interesting consequence of these findings is that immune costimulation is also a metabolic costimulation. Upon T-cell activation, glutaminolysis also increases, whereas β-oxidation of fatty acids decreases (35–37). Thus, it is likely that both glycolysis and glutaminolysis contribute to T cell-dependent production of intra-tumoral lactate.

B lymphocytes have been largely neglected as tumor-infiltrating immune cells. However, there is evidence that they play an important role in antitumor immunity, as antigen presenting cells, as well as a source of cytokines. Their presence within tumors has been considered as a positive prognostic factor in breast cancer (38, 39). Metabolically, B cells are distinct from T cells because, upon activation, they do not preferentially switch from OXPHOS to glycolysis, but rather B lymphocytes use both pathways. However, inhibition of glycolysis or B cell-specific deletion of GLUT-1 suppresses antibody production *in vivo* (35); hence, it is likely that some tumor-infiltrating B lymphocytes contribute to intra-tumoral lactate production.

Overall, the contribution of lactate produced by immune cells to the tumor microenvironment is relatively modest, given that it is dependent on the number of immune cells recruited, their differentiation or activation status, and whether immune cells are dysfunctional because of the immunosuppressive mechanisms developed by the tumor.

## Cell to Cell Transfer of Lactate, the Monocarboxylate Transporters

Monocarboxylate transporters (MCTs) catalyze the proton-linked transport of monocarboxylates (e.g., lactate, pyruvate, and ketone bodies) across the plasma membrane (40–43). MCTs have been found in the plasma membrane of various cell types, including tumor cells (44), erythrocytes (40), and neutrophils (45). MCTs 1–4 isoforms have distinct substrate affinities and have been characterized in detail (46), MCTs are necessary for lactate input into cells that use lactate as an oxidative metabolite (such as skeletal muscle and heart) or cells that use lactate as a substrate for gluconeogenesis (liver). MCT1 is expressed at low levels in most tissues, the expression of MCT2 and MCT3 is restricted to certain tissues; MCT2 is primarily expressed in liver, kidney, and neurons, whereas MCT3 is expressed by the basolateral retinal pigment epithelium and the choroid plexus. High levels of MCT4 are found in white skeletal muscle fibers and at lower levels in other tissues, such as testis, lung, and placenta. Certain cell types, such as chondrocytes, leukocytes, and astrocytes, also express MCT4 (46).

Monocarboxylate transporters of different affinities direct both the influx and efflux of lactate across the plasma membrane. Accumulation of lactate and the acidification that arises in the intracellular milieu might have deleterious consequences for the cell; this is prevented by the cotransport of both protons and lactate by MCTs out of the cells. Of note, transport of lactate relies on the intracellular versus extracellular concentration of lactate, the pH, and the concentration of other substrates of MCTs (47, 48).

Lactate, released by glycolytic cells, such astrocytes, can be transported to other cells that undergo oxidative metabolism, such as neurons (49). This vectorial transport of lactate is mediated by the cell-type specific expression of MCT molecules. This phenomenon is known as the “lactate shuttle” (50, 51). It has been proposed that, in some types of cancer, a similar phenomenon may occur, and this has been referred to as metabolic symbiosis (52).

Several types of human cancer, such as glioma, breast, colorectal, gastric, cervical cancer, and neuroblastoma show increased expression of MCT1 and MCT4, which has been associated with a poor prognosis (48, 53–55).

Cancer stem cells (CSCs) have been involved in tumor recurrence and distant metastasis. CSCs are partially resistant to conventional chemo- and radio-therapies; thus, finding new treatments that can target CSCs may be critical for improving patient survival. Expression of MCT1/2 appears to be important for “stemness” in tumor cells. Curry et al. (56), showed that highly proliferative basal stem cell layer of normal oral mucosa is specialized for the use of mitochondrial fuels, such as lactate and express high levels of MCT1. A similar phenomenon was observed on the highly proliferative, poorly differentiated head and neck squamous cell carcinoma cell population, where the cellular distribution of proliferative markers (Ki-67) correlated with MCT1. The authors suggest that MCT1 may be a novel stem cell marker. Also, 3D spheroids (containing a cell population enriched in cancer stem cells) from ER-positive breast cancer cell lines are sensitive to therapeutic targeting of MCT1/2, via inhibiting the uptake of mitochondrial fuels (ketone bodies and lactate) (57). On the other hand, stem-like CD133-positive fractions from glioblastoma cultures express significantly higher levels of MCT4 mRNA compared with the CD133-negative fractions. *In vitro*, MCT4 silencing resulted in significant growth inhibition and the induction of apoptosis in neurospheres. *In vivo*, MCT4 silencing slowed glioblastoma intracranial xenograft growth. Thus, proliferation and survival of glioblastoma stem-like cells are dependent on the expression of MCT4 (58).

## Effect of Lactate on the Tumor Microenvironment

The tumor microenvironment is an intricate network of extracellular matrix molecules, soluble factors and cells, including stromal cells and adipocytes. Tumor stromal cells include cancer-associated fibroblasts (CAFs), tumor endothelial cells (TECs), and immune inflammatory cells such as macrophages. Stromal cells generate a tumor microenvironment in constant change as tumors invade normal tissues and subsequently seed and metastasize. Among the soluble factors present in the tumor microenvironment, lactate is of particular importance given its effects on cancer and stromal cells. As a consequence of the Warburg effect, cancer cells secrete large amounts of lactate to the extracellular microenvironment, which in turn lowers extracellular pH to 6.0–6.5 (59). Lactate contributes to acidosis, signals for angiogenesis, acts as a cancer cell metabolic fuel, and induces immunosuppression (60–62). Several reports demonstrate that acidosis leads to loss of the T-cell function of human and murine tumor-infiltrating lymphocytes; the T-cell function can be restored by buffering the pH at physiological values (60, 63, 64).

The acidic microenvironment acts as a trigger for pain in cancer patients (65). In addition, lactic acidosis may contribute to the metastasis of some cancers (66). Lactic acidosis induces production of matrix metalloproteinase-9 (MMP-9) in mouse B16 melanoma (67), VEGF-A in glioma and glioblastoma cells (68, 69), and IL-8 expression in pancreatic adenocarcinoma (70, 71) and ovarian carcinoma cells (72), all making the tumor microenvironment even more complex.

Lactate *per se* stimulates angiogenesis, through the activation of the VEGF/VEGFR2 signaling pathway (73, 74), and activates endothelial cells through MCT-1; which triggers the phosphorylation/degradation of IkB**α**, stimulating the NF-kB/IL-8 (CXCL8) pathway that drives cell migration and tube formation (75).

CAFs contribute to tumor survival by several factors including changes in cell metabolism, in which lactate plays a central role. Pavlides et al. (76) formulated the “Reverse Warburg effect” hypothesis, which proposes that tumor cells induce aerobic glycolysis in CAFs. In turn, these cells secrete lactate and pyruvate, which are consumed by tumor cells to undergo Krebs cycle and OXPHOS, resulting in ATP production and a higher proliferative capacity. In consequence, tumor cells can adapt to rapid changes in the tumor microenvironment through reprograming stromal cells and by the metabolic interplay between oxidative (OXPHOS) and glycolytic cells (76, 77).

## Metabolic Symbiosis, Lactate as a Metabolic Substrate

Some authors have suggested that rather than using lactate as a nutrient, cancer cells generally export lactate, which then acidifies the tumor environment affecting stromal cells (48, 78). However, lactate is fundamental for a symbiotic process where tumor cells that grow under hypoxic conditions increase the expression of the glucose transporter GLUT-1 and in consequence the uptake of glucose. This process enhances the glycolytic flux of carbon and the production of lactate, which is then secreted via MCT4. By contrast, tumor cells growing under aerobic conditions take up lactate by MCT1; then, it is converted into pyruvate by the LDH-B, pyruvate enters the Krebs cycle and its products can be used by the OXPHOS pathway for energy production (10). Summarizing, lactate is released in the hypoxic tumor cell compartment, which fuels oxidative metabolism of the aerobic tumor cell compartment, sparing glucose supply to be preferentially consumed by hypoxic cells (52, 79).

The concept of metabolic symbiosis in tumors implies that there is a net flux of lactate (as a metabolic substrate) from hypoxic tumor cells to oxygenated tumor cells, following an oxygen gradient, i.e., it is related to the distance each cell is away from functional blood vessels within the tumor architecture (52, 79).

Inhibition of MCT1 in aerobic tumor cells leads to a higher glucose than lactate consumption, breaking the metabolic symbiosis, at this point the anaerobic tumor cells die from glucose deprivation. This phenomenon was demonstrated in a murine model in which the treatment of lung cancer with an MCT1 inhibitor indirectly induced death of distant hypoxic tumor cells. MCT1 expression has been exclusively found in aerobic regions of human tumor tissue from head, neck, breast, and colon cancers (52). All these results are consistent with the over-expression of LDH-B and the use of lactate as an energy substrate for tumors.

The current model of metabolic symbiosis, considered as a constant environment where a hypoxic core uses glucose and a highly vascularized edge consumes lactate as substrate, should be seen as a dynamic process, because during tumor development and as a result of neovascularization, the pattern of well-perfused and hypoxic areas may change constantly (10, 52). Furthermore, a sudden decrease in the availability of oxygen or glucose may force tumor cells to find an alternate source of energy, such as lactate, for survival during the starving period. Thus, lactate may be used as an alternative to fuel oxidative tumor cells, in which amplification of mitochondrial metabolism contribute to human tumor formation and cancer progression (56, 57). Furthermore, lactate indirectly promotes the survival of hypoxic tumor cells located far from the newly formed blood vessels (10, 80).

## Lactate Effect on Tumor-Infiltrating Immune Cells: A Regulator of Antitumor Immune Response?

The tumor microenvironment can present zones with lactate concentrations of up to 40 mM, which tumor cells can cope with (81). The question now is how do infiltrating immune cells cope with a lactate-rich microenvironment?

To start with, activated immune cells are also lactate producer cells, as it has been demonstrated that, upon activation, T lymphocytes increase the expression of glucose transporters, key glycolytic enzymes, glycolysis rate and, therefore, lactate production (33). Macrophages are also lactate-producing cells; MCT4, which is involved in lactate secretion, is up-regulated by TLR2 and TLR4 agonists in a variety of macrophages; whereas LPS, a TLR4 agonist induces the expression of key glycolytic enzymes, i.e., hexokinase 2 and 6-phosphofructo-2-kinase/fructose-2,6-biphosphatase 3. These enzymes are diminished in macrophages in which MCT4 has been knocked down, suggesting that MCT4 up-regulation represents a positive feedback mechanism in macrophages, which maintains the high glycolytic rate that is required for a fully activated inflammatory response (82).

There is evidence of a deleterious effect of high concentrations of lactate on the tumor-infiltrating immune cells. Clinical evidence supports the possible link between lactate metabolism and limited immune cell infiltration in renal cell carcinoma (RCC). Up-regulation of GLUT-1 expression in RCC biopsies negatively correlates with CD3^+^, CD8^+^ and granzyme B^+^ T cells compared to normal primary cells or kidney tissue. Interestingly, LDH-5 expression in tumor cells, which is one of the five LDH isoenzymes and plays an important role in promoting anaerobic glycolysis, has a negative impact on infiltration by CD3^+^ T cells, but not on CD8^+^ granzyme B^+^ or FOXP3^+^ T cells. These observations suggest that, in addition to the high glucose uptake by tumor cells, lactate also modulates T cells in the tumor environment (83).

Lactate secreted by tumor cells impairs the cytolytic functions of T cells *in vitro*, in particular those of CD8^+^ T cells. Lactate inhibits proliferation and cytokine production of human cytotoxic T lymphocytes (CTLs) by 95%, whereas their cytotoxic activity is inhibited by 50%. Interestingly, a recovery period in lactic acid-free medium restores the CTL function (60). From a series of experiments in which CTLs were treated with 20 mM lactic acid, HCl, or sodium lactate, besides an inhibitor of MCT1 (α-cyano-4-hydroxy-cinnamic acid) or lactic acid derived from melanoma cells, the authors concluded that high lactic acid concentrations in the tumor environment block the export of lactic acid by T cells, thereby disturbing their metabolism and function (60). Lactic acid production by melanoma cells inhibits TAA-triggered IFN-γ production by specific CTLs in melanoma spheroid cocultures (84). Another study also confirmed that lactic acidosis inhibits TCR-triggered cytokine production (IFN-γ, TNF-α, IL-2) and induces partial impairment of lytic granules exocytosis in CTLs. This effect was found to selectively target downstream signaling pathways of the MAPKs p38 and JNK/c-Jun as observed by reduced phosphorylation when CTLs were stimulated in the presence of lactic acid (61).

Tumor-derived lactate is up-taken by tumor-associated macrophages (TAMs) through their MCTs active transporters on the cell membrane, leading to the transcription of the vascular endothelial growth factor (VEGF) and the l-arginine-metabolizing enzyme arginase-1 (ARG1) genes (26, 85). ARG1 hydrolyzes l-arginine to l-ornithine and urea. ARG1 is expressed in myeloid cells, including TAMs, and can support tumor growth and suppresses antitumor immune responses. Lactic acid has been shown to increase ARG1 expression in macrophages, inhibiting T-cell activation and proliferation (86). Recently, Colegio et al. (26) showed that in macrophages cultured under normoxia, HIF1α is stabilized by lactate leading to the transcription of ARG1 and VEGF genes. Lactic acid also favors tumor growth by polarizing macrophages to an M2-like state, a subset with a role in inflammation resolution and tissue remodeling. It remains to be explored whether arginase or VEGF are up-regulated in other myeloid cells that are known to express these enzymes, such as plasmacytoid DCs and myeloid-derived suppressor cells.

Lactic acid transiently inhibits the expression of most LPS-induced genes, this inhibitory effect is not observed after incubation with sodium lactate, and it is attenuated in acidified samples. In monocytes, lactic acid targets are TNF, NF-kB, PTX3, which are down-regulated, and IL-23, which is up-regulated. Also, expression of chemokines (e.g., CCL2 and CCL7) is transiently down-regulated. These effects are mediated by delayed LPS-induced phosphorylation of AKT and the degradation of IkB (87). Thus, lactate modifies monocytes function and consequently contributes to immune suppression within tumors.

Tumor infiltration by mature DCs confers immune activation. However, tumor cells suppress DCs function or otherwise alter the tumor microenvironment so that immune-suppressive DCs are recruited (22). Tumor cells-derived lactate inhibits the differentiation from monocytes to DCs and inactivates the release of cytokines from differentiated DCs (88).

Lactate rather than oxygen availability is responsible for the differentiation to tolerogenic DCs, as exemplified by the increased production of IL-10 and loss of IL-12, in response to TLR stimuli (89). Besides, high extracellular lactate concentration in the tumor microenvironment prevented lactic acid export from glycolytic DCs, thus leading to lactate accumulation and tolerogenic DCs (90).

Recent studies support the notion that the activity of NK cells is inhibited by tumor-derived lactate or low extracellular pH (62, 91). Purified human NK cells cultured in the presence of lactate for 72 h and tested for cytolytic activity against K562 cells exhibited a significant decrease in their cytotoxic activity. This effect was mediated by down-regulation of the NK activation receptor, NKp46 (91).

Tumor cells can secrete anti-inflammatory cytokines, and immunosuppressive cell populations can be recruited to the tumor microenvironment, both directly inhibiting immune responses (92, 93). In addition, recent evidence shows that, for instance, glioblastoma cells secrete enzymatically active LDH-5 that induces the expression of NKG2D ligands on myeloid cells, particularly MICB and ULBP-1 mRNA in healthy monocytes, subverting antitumor immune responses (94). The isoenzyme has the highest efficiency to catalyze pyruvate conversion to lactate (95). Several reports have shown that cancer patients with elevated levels of LDH in sera have a poor prognosis (48, 96, 97) perhaps as a consequence of larger tumor burdens or hypoxic tumor cells with high glycolytic metabolism linked to radio- and chemo-resistance. The secretion of LDH might also contribute to the immune evasion of tumor cells by the induction of NKG2D ligands on host myeloid cells (94).

Lactic acid has been suggested to be a proinflammatory mediator that activates the IL-23/IL17 pathway. Lactic acid increases the expression of IL-23p19 in tumor-infiltrating immune cells activated via TLR stimulation and also induces the Ag- and IL-23-dependent secretion of IL-17 in splenocytes. The activation of the IL-23/IL-17 pathway promotes local inflammatory responses by polarizing immune responses toward a Th17/Th23 profile, which favors the incidence and growth of tumors (98). Taken together, these observations show that lactate plays an important immunoregulatory role in cancer.

## Lactate Receptor-Induced Intracellular Signaling

The l-lactate receptor GPR81 (or hydroxycarboxylic receptor 1, HCA1) was initially classified as an orphan receptor in a search of new G protein-coupled receptors (GPCRs) (99). In 2008 and 2009, it was shown that l-lactate is a natural ligand and agonist of GPR81, along other monocarboxylates such as alpha-hydroxybutyrate, glycolate, alpha-hydroxyisobutyrate, and gamma-hydroxybutyrate (100–102).

The GPR81 receptor has been found in adipocytes (103), in the brain (104), in liver, skeletal muscle, and other human, mouse, and rat tissues (101); and more recently in colon, breast, lung, hepatocellular, salivary gland, cervical, and pancreatic carcinoma cell lines, as well as in tumors resected from patients with pancreatic cancer; in fact, 94% of the pancreatic tumors examined expressed high levels of GPR81 (105).

Roland et al. (105) have shown that shRNA-mediated silencing of GPR81 leads to cancer cell death in culture conditions of low glucose and lactate supplementation, in contrast to cells growing in glucose-containing medium, where GPR81 silencing has no effect. Interestingly, the same authors observed that lactate stimulation of wild-type GPR81^+^ cells induced the expression of genes involved in lactate metabolism, including MCTs, and that, *in vivo*, GPR81 expression levels correlates with the rate of pancreatic cancer tumor growth and metastasis (105).

Whether GPR81 binding to lactate in tumor cells initiates an intracellular signaling pathway or whether the biological effects of lactate on tumor cells are due to lactate uptake, and lactate metabolism remains to be analyzed. The finding that lactate stimulation of GPR81 induces the expression of lactate metabolic genes suggests that GPR81 engagement prompts a cell signaling process.

In this regard, lactate has been proposed to be a signaling molecule in the brain, which is involved in neuronal plasticity; because lactate stimulates the expression of synaptic plasticity-related genes such as Arc, c-Fos, and Zif268 in neurons through a mechanism involving NMDA receptor activity and its downstream signaling cascade Erk1/2, along with an increase in intracellular calcium (104). Lactate also increases intracellular levels of NADH, thereby modulating the redox state of neurons (106).

It is likely that the GPR81-lactate engagement in tumor cells initiates similar signaling pathways. We hope that its characterization will open the possibility of a GPR81-targeted therapeutic intervention in cancer.

## Lactate: A Therapeutic Target in Cancer?

High concentrations of lactic acid in the tumor environment block lactic acid export by T cells, thereby disturbing their metabolism and function. This has led to the suggestion that targeting tumor cell glycolysis and therefore lactic acid production is a promising strategy to enhance antitumor immune responses (60).

Targeting specific MCTs would induce apoptosis of tumor cells due to intracellular acidosis (lactate accumulation) or would inhibit lactate uptake by aerobic tumor cells, thus reducing tumor angiogenesis, invasion, metastasis, and the deleterious effects of extracellular lactate on the immune cells.

There is evidence that MCT4 inhibition can induce accumulation of intracellular lactic acid and the subsequent cell death in hypoxic tumor cells (107). Knockdown experiments have shown that MCT4 is also needed for migration and invasion of MCT4-expressing tumor cells (108, 109), but what about tumor cells growing in normoxic conditions? Would it be possible to block lactate uptake by targeting MCT1, thus disrupting metabolic symbiosis between tumor cells? Lonidamine, an MCT inhibitor, induces an immediate decrease in intracellular pH in neuroblastoma cell lines (Sk-N-SH, CHP134, IMR32, and NGP), which correlates with diminished cell viability within 48 h of treatment (53); knockdown of MCT1, or inhibition of MCTs with the small molecule α-cyano-4-hydroxy-cinnamate, blocks cell proliferation, and migration, and induces apoptosis in glioblastoma cells (110–112). Besides, an inhibitor of MCT1 (AZD3965) has been shown to inhibit small cell lung carcinoma cell lines *in vitro* as well as in an *in vivo* model (113). AZD3965 is undergoing Phase I clinical trials for prostatic and gastric cancer, as well as for diffuse large B-Cell lymphoma (NCT01791595) (114).

In addition, *in vitro* siRNA knockdown of MCT1 and MCT4 in basal-like breast cancer cells in both normoxic and hypoxic conditions led to a decrease in tumor cell aggressiveness, concomitant with decreased lactate transport, cell proliferation, migration, and invasion. MCT-knockdown inhibited tumor formation and growth in a model of tumor xenografts in nude mice (107).

Targeting lactate production and lactate transport are promising therapeutic strategies for cancer. Despite their success, current MCTs inhibitors are not selective (48). For example, α-cyano-4-hydroxy-cinnamate also inhibits the chloride-bicarbonate exchanger AE1 (112) and the mitochondrial pyruvate carrier (115). Lonidamine inhibits the hexokinase 2 enzyme activity *in vitro* (116). Therefore, it seems worthwhile to develop new potent small molecules to selectively inhibit the various MCTs involved in tumor growth.

The recent discovery of potent and specific MCT1 inhibitors developed by Astra-Zeneca confirms that MCTs could be promising pharmacological targets including their use for cancer chemotherapy (48, 115, 117). However, these inhibitors were originally described to prevent proliferation of T lymphocytes. Thus, the effect of inhibition of MCT1 on tumor-specific T cells remains to be seen.

Targeting tumor metabolism via anti-glycolytic therapies has been proposed as an attractive therapeutic approach, as glycolysis is a key converging node for multiple signaling pathways in cancer cells. LDH-A, the enzyme that converts pyruvate to lactate, is currently a promising target (59).

On the other hand, 3-bromopyruvate (3-BrPA), a drug under development, has cytotoxic effects and decreases cellular energy levels by inhibiting glycolysis. 3-BrPA is probably best characterized as a toxic molecule rather than a specific inhibitor of glycolysis or MCT1. It has been proposed as a therapeutic alternative to deliver toxic molecules to glycolytic tumors by using the MCT1-mediated transport (118).

Studies performed on LDH-A-suppressed cancer cell lines show that these cells exhibit reduced tumor progression in xenograft models, due in part to the increased production of reactive oxygen species (ROS) and cell death, as a result of increased cell respiration (59). Metabolic reprograming in lung tumor cells, following LDH-A abrogation, reduces lactate production concomitant with an increased flow of carbon from both glucose and glutamine through the Krebs cycle, oxygen consumption, and mitochondrial ROS production (59, 119).

Several small-molecule LDH-A inhibitors are being tested for their anticancer activity. However, many of them still show low selectivity and potency (48). Interestingly, a new LDH-A inhibitor is capable of suppressing cancer stem cell function, a type of cells which are not targeted by most current therapies for cancer (59).

## Lactate: A Prognostic Biomarker in Cancer?

The possible role of lactate as a predictive biomarker of overall survival in cancer patients arises from several studies that lactate intra-tumoral levels are inversely correlated with overall and disease-free patient survival, as reviewed by Hirschhaeuser et al. (120). Sandulache et al. (121) recently reported that lactate may be a quantitative biomarker of acute radiation response. The authors showed in a murine model that irradiation of tumors triggered a rapid, dose-dependent, transient decrease in lactate levels. Acute lactate perturbations after irradiation could identify hypoxic conditions and correlated with hypoxia-induced radioresistance. Recently, Blatt et al. (122) showed that, in a cohort of head and neck squamous cell carcinoma patients, high lactate levels in tumor tissue are inversely correlated with the overall and recurrence-free survival after surgery and radiation during a 15-year follow-up.

## Concluding Remarks and Perspectives

**Figure 2:**
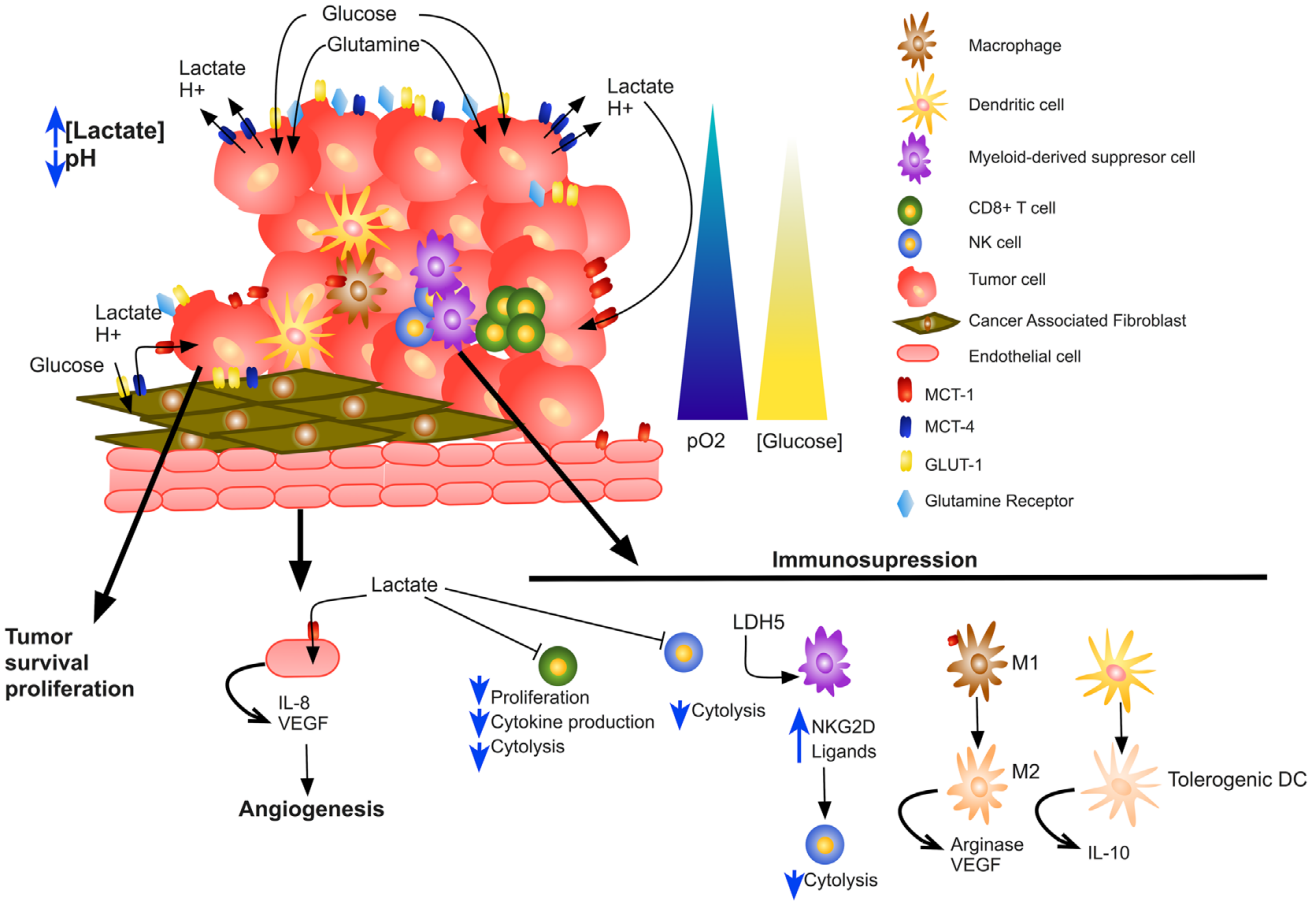
**Impact of lactate on tumor microenvironment**. Increased lactate secretion by tumor and stromal cells acidifies the tumor microenvironment, increases tumor cell survival and proliferation, stimulates angiogenesis, and results in skewed immune response by altering several immune infiltrating cells.

In this review, we present an integral view of the role of lactate in tumor progression. Tumor cells take advantage of the metabolic symbiosis, which is not only stablished among tumor cells; in addition, stromal cells participate in a “Reverse Warburg Effect,” where fibroblasts may produce lactate that normoxic tumor cells consume to produce energy. Moreover, several tumor-infiltrating immune cells contribute, in some extent, to the total amount of lactate within the tumor. Lactate is not only consumed by tumor cells for their survival, but it also stimulates angiogenesis. What is more, lactate has an immunosuppressive role, affecting several immune cell functions such as T-cell proliferation, cytokine production, and cytotoxic activity of NK and CD8^+^ T cells (Figure 2).

Taking all above into account, lactate is needed not only for survival but also for promoting tumor growth, and it seems now a promising target for cancer therapy. Although considerable progress has been made in the last few years finding novel inhibitors for both the MCTs and the LDH, more investigations on the role of lactate will continue to provide important new insights into the molecular interactions between lactate and tumor growth identifying new targets for cancer therapy.

## Author Contributions

SR-G contributed to manuscript writing, reviewing, and designed the figures. MMM-A contributed to manuscript writing and reviewing. HP-G designed, coordinated the review, and contributed to manuscript writing. FS-G conceived the review and contributed to manuscript writing. All authors approved the final manuscript.

## Conflict of Interest Statement

The authors declare that the research was conducted in the absence of any commercial or financial relationships that could be construed as a potential conflict of interest.
